# A machine learning approach based on ACMG/AMP guidelines for genomic variant classification and prioritization

**DOI:** 10.1038/s41598-022-06547-3

**Published:** 2022-02-15

**Authors:** Giovanna Nicora, Susanna Zucca, Ivan Limongelli, Riccardo Bellazzi, Paolo Magni

**Affiliations:** 1 Department of Electrical, Computer and Biomedical Engineering, University of Pavia, Pavia, Italy; 2 enGenome S.R.L., Pavia, Italy

**Keywords:** Biomedical engineering, Data mining, Genetic testing, Genetic variation, Personalized medicine, Genetic predisposition to disease, Genetic counselling

## Abstract

Genomic variant interpretation is a critical step of the diagnostic procedure, often supported by the application of tools that may predict the damaging impact of each variant or provide a guidelines-based classification. We propose the application of Machine Learning methodologies, in particular Penalized Logistic Regression, to support variant classification and prioritization. Our approach combines ACMG/AMP guidelines for germline variant interpretation as well as variant annotation features and provides a probabilistic score of pathogenicity, thus supporting the prioritization and classification of variants that would be interpreted as uncertain by the ACMG/AMP guidelines. We compared different approaches in terms of variant prioritization and classification on different datasets, showing that our data-driven approach is able to solve more variant of uncertain significance (VUS) cases in comparison with guidelines-based approaches and in silico prediction tools.

## Introduction

The interpretation of genomic variants is crucial for the diagnosis and risk assessment of a broad set of diseases, from rare Mendelian to hereditary cancer^1^. Thanks to next generation sequencing (NGS) technologies, thousands of variants can be identified for a single patient depending on the type of experimental assay (e.g., gene panel, whole exome or genome). In the context of monogenic inherited disorders, only few of these variants might be pathogenic for the condition of interest^2^. In order to identify them, clinical laboratory geneticists often leverage on in silico functional tools that are able to assess protein variant intolerance, splicing or gene regulatory alterations^3^. Several of these prediction tools with different characteristics and scope have been developed so far^4–7^.

Although these approaches quantitate the potential damaging effect of a variant on the gene product, they are less reliable in pathogenicity evaluation, since a disruptive variant might not be implicated in a disease^1^: studies have shown that up to one third of benign variations could be predicted as disease causing^8^ and that even state-of-art in silico prediction tools should not be used as a standalone evidence to infer variant pathogenicity^9^. Indeed, other evidence types, from family history to literature and phenotype matches need to be collected.

To convey a standard procedure for the integration of all different sources of information in a variant interpretation pipeline, the American College of Medical Genetics and Genomics (ACMG), along with the Association for Molecular Pathology (AMP) published their guidelines in 2015^1^. The proposed categorization rules leverage different types of evidence and standardize interpretation into five different classes: “Pathogenic”–“Likely pathogenic”–“Benign”–“Likely benign”–“Variant of Uncertain Significance (VUS)”. Twenty-eight criteria are defined by the ACMG/AMP guidelines to describe each variant with respect to different characteristics (such as population allele frequencies or family history) and with respect to different degrees of pathogenicity. ACMG/AMP criteria are hierarchically organized in groups of different levels of evidence to support pathogenic or benign classification. The ACMG/AMP-based classification workflow can be viewed as a two steps procedure: first, each variant is characterized by the set of criteria, then the number of criteria across different levels of evidence is evaluated by the IF–THEN rules to output the classification.

Since their publication, the ACMG/AMP guidelines have been spreading in the clinical practice^10^, they have been specialized to different genes^11–13^ and several tools have been proposed to automate their application^14–18^.

However, because of the heterogeneous information that feeds ACMG/AMP guidelines, often joined with lack of data (e.g., family segregation, functional studies), these systems are usually not able to apply all the needed criteria. VUS variants may be implicated in disease development, and their uncertain interpretation reflects a lack of sufficient or conflicting information. Within this context, it is essential to develop new methodologies to improve upon the granularity of the ACMG/AMP guidelines to bring more certainty to classifications.

In order to facilitate the pathogenicity assessment of a list of variants, which usually includes several VUS variants, tools that assign a quantitative measure rather than solely the guideline class have been proposed^19–21^.

CardioVAI^19^ and CharGer^20^ associate to each variant a score that depends on the number and level of evidence of the applied ACMG/AMP criteria. Another approach models the ACMG/AMP guidelines within a Bayesian framework^21^, thus providing a probabilistic score of pathogenicity.

These approaches can therefore be used to stratify VUS, i.e., by highlighting the most interesting ones that might need a further evaluation of the supporting evidence and, conversely, deprioritizing VUS with few or none supporting evidence of pathogenicity. These approaches can finally contribute in mitigating the VUS impact that still remain the major issue in diagnostic variant interpretation^22,23^.

However, as the rate of interpreted variants from molecular laboratories increases and these classifications within their supporting evidences are made publicly available in repository such as ClinVar^24^ or in curated benchmark datasets^3,25^, data-driven approaches can be used to gain knowledge from this amount of data to support interpretation of previously unseen variants. Machine Learning (ML) approaches, from Logistic Regression (LR) to Tree-based techniques, have already been applied to distinguish pathogenic from benign variations in the last years^26–28^.

The success of a ML algorithm in classification and prioritization depends on the robustness of the training set and on the completeness of the features that describe each instance to be classified (in our case, genomic variant). The training set is a subset of instances (variants) with known class (pathogenic or benign) coming from an underlying true population that is actually unknown or inaccessible. This set of variants known to be benign or pathogenic are therefore seen as a reference for ML algorithms, that will learn from it patterns which can be invoked for the classification of new and previously unseen variants.

The second important aspect in the development of a ML approach is the choice of attributes, or features, that must be selected to characterize each variant. Researchers and clinicians already depict genomic variants based on their effect on transcription or translation, their genomic context and on several annotation resources publicly available^29^. Along with the pathogenicity prediction based on in silico approaches mentioned above, population allele frequency or evolutionary information have been selected as features for ML algorithms^26–28^.

ACMG/AMP criteria can be considered the most promising high-level features, well characterized by the genetic experts. Their adoption as attributes to prioritize variants with a Gradient Boosting approach has been recently described^30^. In such method, 15 original ACMG/AMP criteria have been used as features to train the model, in combination with phenotypic, gene level and other functional variant data. This choice however has two limitations: first, by taking into account a subset (15 out of 28) of the ACMG/AMP criteria, the method cannot represent the full spectrum of ACMG/AMP evidence and it is not robust to gene-specific ACMG/AMP guidelines that dismissed some of the criteria or emphasized others; second, it could undermine the contribution of ACMG /AMP criteria to the final classification, resulting in possible conflicting interpretation with guidelines itself.

Here, a method for variant prioritization that is both data-driven and guidelines-based is presented. ACMG/AMP criteria are used to train a ML classifier on well-known pathogenic and benign variations. The idea behind is that ACMG/AMP levels of evidence, rather than criteria themselves, can be exploited as features for the ML method, which in turn can provide a probabilistic score of pathogenicity. This score can be used to rank VUS variants, thus helping in pinpointing those disease-causing variants that remain VUS possibly because not every evidence might be automatically assessed for lack of data. With this approach, the method is able to be potentially trained and applied on variant dataset with sparse and even gene-specialized ACMG/AMP criteria, because the level of evidences (features) of the different criteria have been a priori established by the genetic experts at the time of the guidelines drafting. Our method was trained on variants associated with a broad spectrum of disorders, and therefore it can be applied to any type of diseases, from cardiovascular diseases to cancer.

To investigate the contribution of additional low-level information, another ML model that leverages on different variant and gene annotation features was trained and compared.

The developed ML approaches were evaluated in terms of classification performance (i.e. the ability to correctly discriminate pathogenic and benign variants) and prioritization (i.e. the ability to prioritize true pathogenic variants) on real variant data^2^. Classification and prioritization of the proposed ML approaches were compared with classification and prioritization performed by guidelines-based scoring methodologies (the pathogenicity score suggested in Nicora et al.^19^ and the Bayesian modeling suggested in Tavtigian et al.^21^^)^ and with two widely used in silico scoring methods, VVP^31^ and CADD^32^.

Our approach was built using Python 3 and the scikit-learn package for Machine Learning implementation^33^. Genomic variant data exploited in this study for training and evaluation are provided upon request. Source code to reproduce results reported in this paper is available at https://github.com/GiovannaNicora/MLVar.

## Methods

### Variant datasets

ML algorithms learn from a set of data, known as training set. In our case, ML learns to distinguish between benign and pathogenic variants from a training set of known benign and pathogenic variants. To guarantee good performance on new data, it is crucial that such training set is as much as possible representative of the entire domain of interest. After the training phase, the classification ability of the trained ML model is usually tested on one or more independent dataset. This phase is known as validation. To include as much information about known pathogenic and benign genomic variants for training and validation, three different public sources were selected (see Table 1).

**Table 1:** Datasets collected and purpose.

	Dataset name	Purpose	# of variants
Model building	Clinvitae training	Training	8496
	Clinvitae probability threshold tuning (PTT)	Tuning the probability threshold for classification	4247
Model validation	Clinvitae test	Comparison between different ML methods and the pathogenicity score in^19^	1415
	Clinvitae Validation	Testing classification of the selected ML method, in comparison with the pathogenicity score and the bayesian score	161,744
	ICR639	Testing classification and prioritization of the selected ML method on a real dataset, in comparison with the pathogenicity score, the bayesian score, CADD and VVP	18,046

As training set for our ML model, variants reported in Clinvitae database up to 2017 were exploited. Clinvitae was a database gathering clinically-observed genetic variants associated with a broad set of diseases from several public resources, including ClinVar, ARUP Mutation Database, Carver Mutation Database, and Emory Genetics Laboratory Variant Classification Catalog. Interpretation was performed by clinical experts, and results were shared. However, Clinvitae website is no longer maintained and Clinvitae variants can now be found in ClinVar repository, submitted by Invitae (https://www.invitae.com/en/about). The database used in this study was downloaded in October 2019 (see the Data availability statement for further details). We removed all the variants with conflicting interpretations among different sources and those reported by ClinVar submitters. In fact, in our implementation of the ACMG/AMP guidelines, ClinVar is exploited for ACMG/AMP criteria evaluation and triggering^19^. Since the ML model will include ACMG/AMP-based features, we want to prevent our training set to be biased towards ClinVar variants, thus preventing the model to overfit on ClinVar-reported variants. The collected dataset has 5649 confirmed pathogenic variants and 8509 confirmed benign variants. The interpretation provided by Clinvitae submitters in forms of ACMG/AMP class was considered as ground-truth to train and test our models. This dataset has been divided randomly, while maintaining the classes proportion, in 3 different subsets: 60% of variants were used to tune and train the ML algorithm (“Clinvitae Training”), 30% of variants were exploited to select the best probability threshold for classification (“Clinvitae PTT”), while the remaining 10% is exploited for comparing classification performances (see Table 1) (“Clinvitae Testing”).

The trained model was further evaluated on two other independent datasets. First, 161,744 variants (65,626 pathogenic and 96,140 benign) reported in Clinvitae up to 2019 that were not included in Clinvitae Training, Clinvitae PTT and Clinvitae Testing were selected.

Finally, ML performances were tested on real data from 639 individuals that underwent NGS test to detect pathogenic variants in Cancer predisposition genes, such as BRCA1 and BRCA2^2^. VCF files for each donor, along with the list of pathogenic variants reported in this cohort, have been made available to the research community in 2018^2^. About 18.000 Single Nucleotide Variants and short Indels were retained in the analysis, 550 of those were reported as pathogenic, and the remaining ones were assumed to be benign variations. This dataset has been exploited to test both classification and prioritization performances on VCF file. In fact, despite thousands of variants are reported for each patient, up to two are actually pathogenic. Therefore, it is crucial to rank the few pathogenic variants in the very first positions to facilitate variant interpretation.

**Figure 1:**
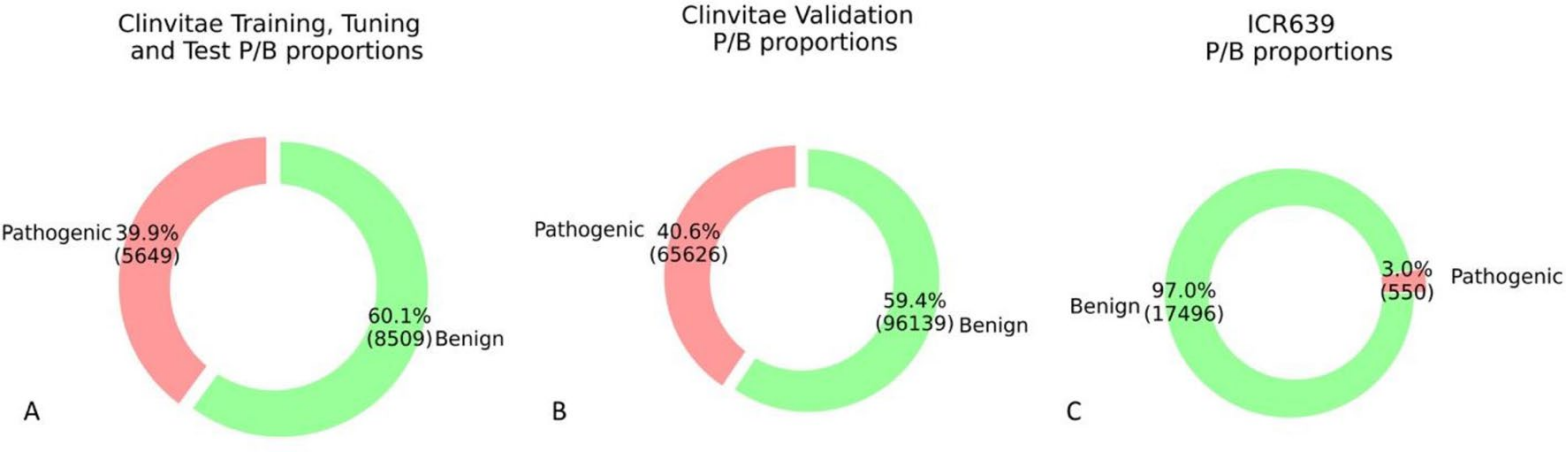
Proportion of benign and pathogenic variants in **(A)** Clinvitae training, PTT and test sets, **(B)** Clinvitae validation set, **(C)** ICR639 hereditary cancer dataset.

The proportions of Pathogenic/Benign variants in each dataset are shown in Fig. 1. The real proportion of pathogenic variants in a cohort of patients is much lower (about 3%) (Fig. 1C) than the one reported in Clinvitae datasets, both for model training and validation (which in both cases is around 40%) (Fig. 1A,B).

### Feature selection

In order to train ML algorithms, two different approaches were applied: the first one characterizes each variant only with ACMG/AMP-based features (named “A” approach), the second one adds also annotation features (“B” approach).

The set of seven ACMG/AMP-based features represent the number of ACMG/AMP triggered criteria in different ACMG/AMP levels of evidence as defined in Richards et al.^1^: “Pathogenic Very Strong” (PVS), “Pathogenic Strong” (PS), “Pathogenic Moderate” (PM), “Pathogenic Supporting” (PP), “Benign Stand-Alone” (BA1), “Benign Strong” (BS) and “Benign Supporting” (BP). For instance, if a variant is both PS1 and PS3 (two “Pathogenic Strong” criteria) the value of the feature “nPS” (i.e., the number of triggered “Pathogenic Strong” Criteria) in our dataset will be 2. If a variant has no “Pathogenic Supporting” criteria, its value of “nPP” (i.e., the number of triggered “Pathogenic Supporting” Criteria) will be 0.

The second set of annotation features includes all features from “A” approach and additional annotation fields, manually selected among all available information, spanning from population allele frequencies to different in silico prediction scores and variant effect on transcripts, that have been collected from public omics resources (see Suppl. Table S1 for the complete list). A previous similar work^30^ directly exploited a subset of ACMG/AMP criteria (such as PS1, PS2, PS3) as Boolean features for ML models. This means that when a variant triggered a particular criterion, the corresponding feature will be set to one. However, this representation considers each ACMG/AMP criterion as independent, without taking into account the relationship among criteria, as defined by the ACMG/AMP levels of evidence. Moreover, this approach does not include all the available criteria (e.g. criteria based on co-segregation). Finally, it does not allow user to modify criteria levels of evidence towards classification, thus preventing the adaptation of this method to disease and gene-specific refinements of the guidelines. For these reasons, we used the aggregation of ACMG/AMP criteria at levels of evidence. Instead of exploiting Boolean features, each corresponding to one ACMG/AMP criterion, we build numerical features corresponding to the number of ACMG/AMP criteria triggered by a variant in a specific level of evidence. In this way, user could also add information about patient or disease-specific ACMG/AMP criteria that could not be implemented in an automatic way.

Both variant annotation and ACMG/AMP criteria triggering was performed using the eVai software (https://www.engenome.com) version 0.7.

The two approaches (A-approach and B-approach) were compared by building two different ML models. First, a well-known ML algorithm, named Logistic Regression (LR), was trained and tested on a variant dataset with ACMG/AMP-based features only (“A” approach). Then LR was trained by using the combination of ACMG/AMP-based features and annotation features (“B” approach). While the A approach has 7 features, equal to the number of ACMG/AMP levels of evidence, the B approach has 53 different numeric and Boolean features.

### ML approach based on logistic regression

In this study, we used as ML algorithm the Logistic Regression (LR). LR was used to compute the probability of pathogenic classification for a genomic variant, given its ACMG/AMP “profile”, that is the number of active criteria in different levels of evidence (“A” approach) or in conjunction with genomic annotation features (“B” approach) (see Supplementary Table S1 for the complete list of features). LR models the posterior probabilities of different outcomes (classes) as a linear combination of instance attributes^34^. LR has been widely applied for prediction in biomedicine^35^, even for genomic variant classification^26^.

Given a binary classification problem, the posterior probability of class 1 is modeled using a logistic function:

$$P\left(1 | x,\beta \right)=\frac{1}{1+{e}^{-\left(\beta \cdot x\right)}}$$

The coefficients $\beta$ are computed through maximum likelihood optimization and different penalty methods could be applied to shrink the $\beta$ estimates, thus preventing overfitting. Shrinkage is usually achieved by imposing a penalty on the size of the estimates. Two commonly used shrinkage methods are the Ridge regression and the Lasso regression. In the Ridge, the $\beta$ coefficients are estimated by minimizing the residual sum of squares, but the penalty function imposes that the $\beta$ ${l}^{2}$ norm must be equal or less a complexity parameter, while the Lasso imposes that the $\beta$ ${l}^{1}$ norm is equal or less the complexity parameter. As a consequence, the coefficients estimated by the ridge are reduced, but never set to zero. Instead, coefficients estimated with the Lasso can assume the value of zero. By estimating (some) of the coefficients as zero, the Lasso automatically performs feature selection, In *scikit-learn* Logistic Regression implementation, shrinkage (or penalty) is an input hyper-parameter of the LR algorithm. The l1 penalty corresponds to the Lasso implementation, while the l2 corresponds to the Ridge. The two different penalties are therefore considered as hyper-parameter of the model. The following pipeline for training, deployment and evaluation of the LR was adopted for both the approaches “A” and “B”, t:

1. The best set of hyper-parameters for the classifier (such as the penalty type, l1 or l2) is selected through a fivefold nested cross validation^36^ performed on the Clinvitae Training Set. Once the parameters are tuned, the classifier is trained on the complete Clinvitae Training Set. Probability calibration is checked through the Brier score ^37^.
2. The trained and eventually calibrated classifier is exploited to predict the pathogenic class probability on the Clinvitae PTT set. Different classification thresholds were considered on this dataset to improve precision (decreasing sensitivity) through an approach based on the computation of the ${F}_{\beta }$ metric^38,39^ (see Supplemental Material). In this analysis, benign/pathogenic classification were weighted with different costs, by changing the threshold for pathogenic classification from the default value (0.5). In fact, given the high number of variants detected for each patient and knowing that only few variants are actually pathogenic in a real scenario, the classification thresholds were set in order to minimize the number of false positive (i.e., benign variants that are classified as pathogenic).
3. The classification performance was evaluated with the best probability thresholds estimated at step 2 on Clinvitae Test dataset, Clinvitae Validation dataset and ICR639 set.

### Guidelines-based scoring methods

Guidelines-based scoring methods convert ACMG/AMP five-tier system into a prioritization system. They can be directly applied just after ACMG/AMP criteria are evaluated because they do not learn from data.

The first tested method, named “Pathogenicity Score” (PS)^19^, is a simple scoring system which assigns to each variant the sum of scores that are proportional to the levels of evidence of ACMG/AMP criteria triggered by the variant itself, according to the deterministic score reported in Nicora et al.^19^.

The second approach transforms the ACMG/AMP guidelines in a Bayesian framework^21^. The posterior pathogenic probability can be easily calculated once two parameters have been set: the prior probability for pathogenicity and the strength of the very strong evidence. Prior was set to 0.1 and the strength equal to 350 as suggested by the original paper^21^. With this setting, when the posterior probability for pathogenic classification is greater or equal to 0.9 than the variant will be considered as pathogenic. In the following, we will refer to the posterior probability as “Bayesian Score” (BS).

Both methods require the assignment of ACMG/AMP criteria to each variant. For this purpose, eVai software was used. eVai performs automatic application of the ACMG/AMP guidelines by integrating several omics resources and implementing variant interpretation rules. ACMG/AMP guidelines implementation details can be found in Nicora et al.^19^ and performance on specific use cases are widely reported in the literature^40–44^. However, any tools that calculates ACMG/AMP criteria, such as InterVar^14^ or CharGer^20^ could be alternatively used. Since our aim is both to compare prioritization and classification performances of guidelines-based methods with ML, a threshold for pathogenicity classification was set. The threshold for PS has been selected according to the ${F}_{\beta }$ approach, applied also for ML on the Clinvitae PTT set. The best threshold to lower the number of false positive based on the Clinvitae PTT was equal to 4. On the other hand a variant has been considered to be pathogenic if its BS is equal or greater than 0.9, as reported in Tavtigian et al.^21^, otherwise it will be considered as benign. This assumption was made in order to obtain a binary classification, even though the Bayesian framework follows the ACMG/AMP five-tier system.

### In silico prediction tools for variant scoring

Two in silico prediction tools (CADD and VVP) were selected to further evaluate their performances in pathogenicity detection and variant ranking. Combined Annotation Dependent Depletion (CADD) combines multiple annotation metrics into a single score, by using a Support Vector Machine trained on high-frequency human derived alleles and simulated data. It was first published in 2014 and since then it has been updated^6^. VAAST Variant Prioritizer (VPP) assigns a prioritization score to a variant based on amino acid substitution, allele frequency and phylogenetic conservation^31^. CADD (version GRCh37-v1.4) and VVP (version 1.5) were used to prioritize ICR639 variants. Thresholds suggested from best practice were adopted in order to classify ICR639 variants as pathogenic or benign according to CADD and VVP scores^31^. For CADD, a variant is considered to be pathogenic if its CADD score is above 20. For VVP, a variant is considered to be pathogenic if its VVP score is above 57.

### Performance evaluation

Classification results are evaluated in terms of different metrics, such as precision, recall and specificity. Prioritization performances are evaluated with a tie-aware version of the Discounted Cumulative Gain^45^. See Supplemental Material for an overview of these metrics.

## Results

### Logistic regression model and feature selection

For each approach (“A” and “B”), a LR model was trained. We will refer to the two trained models as LR-A and LR-B. All model parameters for LR-A and LR-B are reported in the Jupyter Notebook (https://github.com/GiovannaNicora/MLVar).

The LR $\beta$ coefficients estimated with the training process are reported in Table S2 for LR-A and in Table S3 for LR-B. In the “A” approach, the penalty selected in the nested cross validation was the *l2*, therefore, as reported in Table S2, none of the ACMG/AMP-derived features have a null coefficient. Positive values of coefficients mean that the corresponding features, for instance *nPVS*, contributes to the positive (pathogenic) classification, while negative coefficients are associated to features contributing to a negative (benign) classification. For the “B” approach, the best model was the one with *l1* penalty. In this final model, 32 features out of 53 were actually retained. Notably, all the 7 features calculated from the ACMG/AMP guidelines were retained. Half of the population frequency attributes and only 13 out of 30 features encoding the variant effect on the transcripts were retained (Table 3S).

Interestingly, the positive and negative signs of both the LR-A and LR-B estimated coefficients for the ACMG/AMP-derived features match with the associated levels of evidence for pathogenicity: nPVS, nPS, nPM and nPP have positive signs, proving that their values contribute to an increase in pathogenicity likelihood. On the contrary, nBA, nBS and nBP have negative signs, demonstrating that they are associated with an increased likelihood of benign classification (Table S2 and S3).

### Classification performance

First, we evaluated whether there is a significant difference between LR-A and LR-B approaches by a fivefold cross validation performed on the Clinvitae Training set. The accuracy score of LR-A has a mean of 97.84% and 3.8% standard deviation, while for the LR-B the mean is 98% and 4.9% standard deviation. Both approaches have similar performance, therefore we evaluated both of them in the following analysis.

Once trained on the entire training set, the two models are used to predict pathogenic and benign probability classification for each variant in the Clinvitae PTT Set. To attribute a class to each case based on the probability classification, a classification threshold was adopted following the ${F}_{\beta }$ approach described in the Supplemental Material. In particular, choosing a $\beta$ of 0.35, the classification threshold is equal to 0.85 for LR-A, while is 0.83 for LR-B. Variants with probability of pathogenic classification above or equal to these thresholds will be classify as pathogenic, otherwise benign. For instance, if a variant has pathogenic probability equal to 0.8, it will be classified as Benign both for LR-A and for LR-B. The same procedure is used to select the best threshold for classification with the Pathogenicity Score (PS). The best threshold for the Pathogenicity Score (PS) calculated with this approach is 4.

Performance of LR-A and LR-B were compared on Clinvitae Test Set against the simple scoring method PS (Table 2). The threshold for the Pathogenicity Score (PS) is 4.

**Table 2:** Results of logistic regression A approach (LR-A), logistic regression B approach (LR-B) and pathogenicity score (PS) on the Clinvitae test set.

	LR-A	LR-B	PS
Accuracy	0.9752	0.9780	0.9597
Precision	0.9889	0.9926	0.9941
AUC	0.9708	0.9737	0.9505
F1	0.9684	0.9720	0.9472
Recall	0.9487	0.9522	0.9045
Balanced accuracy	0.9708	0.9737	0.9505
MCC	0.9486	0.9546	0.9174
PRC	0.9587	0.9643	0.9374

**Figure 2:**
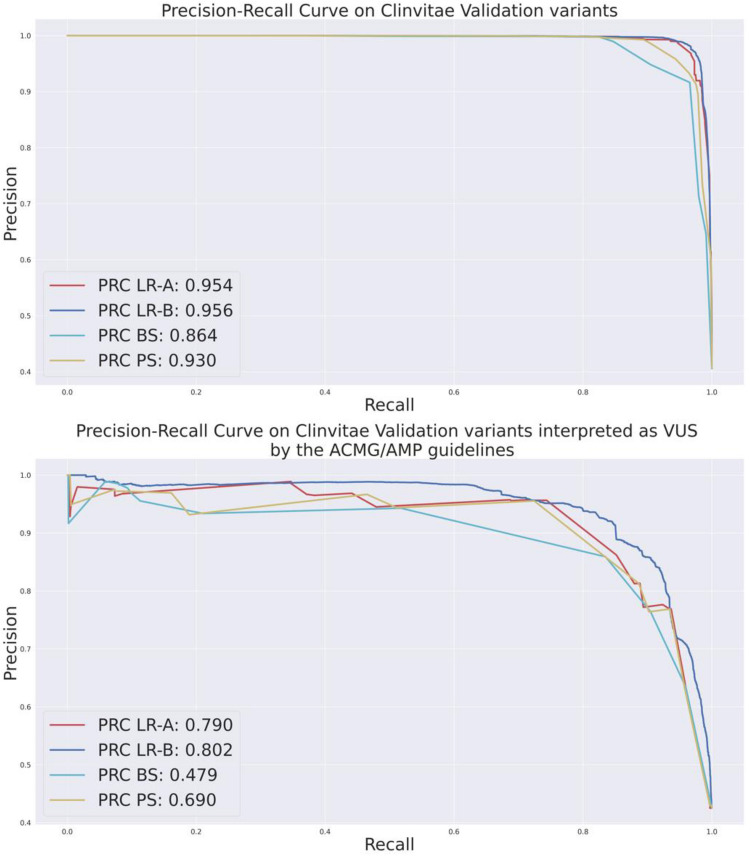
Precision and recall curves for LR-A, LR-B, BS and PS on all Clinvitae validation variants and on Clinvitae validation variants interpreted as VUS according to the ACMG/AMP guidelines.

Performance of LR-A, LR-B, PS and the Bayesian modeling of ACMG/AMP criteria^21^ were then compared on the Clinvitae Validation. Table 3 reports results on the entire dataset (columns “All”) and on the subset of variants that are interpreted as VUS by eVai according to the ACMG/AMP guidelines (columns “VUS”), whereas Fig. 2 reports the Precision-Recall Curves in both cases. The number of variants interpreted as VUS due to conflicting interpretation are 606, while for 28,399 variants the triggered ACMG/AMP criteria were not sufficient to reach a clear classification. Therefore, the total number of VUS is 29,005, representing the 17.93% of the dataset. Among VUS variants, 42.5% are reported as pathogenic in Clinvitae, and the remaining are benign.

**Table 3:** Performance of logistic regression A approach (LR-A), logistic regression B approach (LR-B), pathogenicity score (PS) and the Bayesian approach (BS) on the entire Clinvitae validation set (“all” columns) and on the subset of Clinvitae variants that are interpreted as VUS by the ACMG/AMP guidelines (“VUS”) columns.

	LR-A		LR-B		PS		BS	
	All	VUS	All	VUS	All	VUS	All	VUS
Accuracy	0.9717	0.8630	0.9702	0.8507	0.9559	0.7793	0.9082	0.6161
Precision	0.9901	0.9582	0.9943	0.9703	0.9870	0.9435	0.9979	0.9679
AUC	0.9667	0.8429	0.9641	0.8270	0.9476	0.7443	0.8872	0.5489
F1	0.9643	0.8147	0.9621	0.7921	0.9433	0.6633	0.8727	0.1817
Recall	0.9398	0.7087	0.9318	0.6692	0.9033	0.5114	0.7755	0.1003
Balanced accuracy	0.9667	0.8439	0.9641	0.8270	0.9476	0.7743	0.8872	0.5489
MCC	0.9419	0.7302	0.9542	0.7193	0.9098	0.5738	0.8184	0.2357

Eventually, the four aforementioned strategies were compared with classification made from CADD^32^ and VVP^31^ on the ICR639 Dataset (Supplementary Table S4–S8).

The ability of these tools to solve interpretation for those variants that according to the ACMG/AMP guidelines rules are classified as uncertain was assessed on the ICR639 dataset. Based on ACMG/AMP criteria, 66% (11,862/18,046) of ICR639 variants were classified as VUS: 11,858 of them because none of the ACMG/AMP rules were verified according to their triggered criteria, and four of them benign variants that were reported both as Pathogenic/Likely pathogenic and Benign/Likely benign according to the ACMG/AMP guidelines applied through the eVai software. Among all the VUS variants, only 26 were pathogenic. We focused on classification of VUS variants made by the five approaches (see confusion matrices in Supplementary Tables S9–S14).

VVP were able to identify all 26 pathogenic variants, but with a very high number of false positive (6342), CADD has a lower value of false positive, but it predicts as benign 7 pathogenic variants. The PS score has the best MCC, while BS has a high specificity, but low recall, being able to detect as pathogenic only seven true pathogenic variants. The LRs show the highest balanced accuracy (which is mean between the ability to detect pathogenic variants and the ability to detect benign variants). LR-A correctly identifies 25 out of 26 pathogenic variants, while having 315 false positive, while LR-B identifies 24 pathogenic variants, with three false negative and 188 false positive.

### Prioritization performance

**Figure 3:**
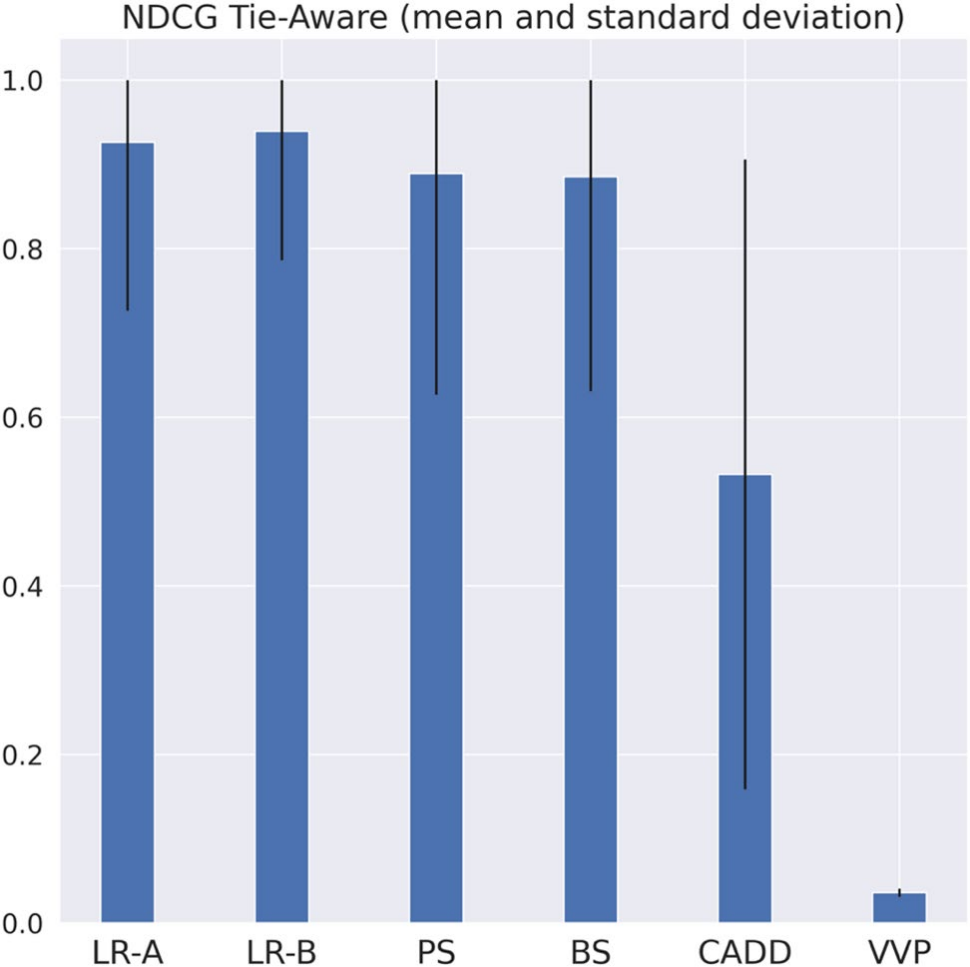
Normalized discounted cumulative gain tie-aware (mean and standard deviation) computed on patients from ICR639 hereditary cancer dataset.

When evaluating the effectiveness of a variant interpretation tool, it is important also to understand its prioritization ability. Good prioritization ability is achieved when the most interesting instances (in our cases, the pathogenic variants) are ranked in the first positions, based on the tool’s output score (such as probabilities). Prioritization is extremely important in variant interpretation. In fact, for each patient, up to thousands of variants need to be interpreted. It is desirable that the most interesting variants are prioritized by the tool, to help clinicians in focusing on few candidate variants. The prioritization performance were evaluated on each patient in the ICR639 dataset, using a tie-aware version of the Normalized Discounted Cumulative Gain (NDCG)^40^. The NDCG is a popular metrics in information retrieval, and it measures the usefulness of an item based on its position in a list. In our scenario, for a patient that undergoes NGS analysis, the list of his/her variants will be ranked based on a pathogenicity prediction score. We want to evaluate how good is a prioritization tool to report the few putative pathogenic variants in the very first positions. A tie-aware version of the NDCG was used, since for some tools, especially for the pathogenicity score, it can happen that many variants have the same score (tied results). For each patient in the ICR639 dataset the NDCG was calculated for each tool (LR-A, LR-B, PS, BS, CADD and VVP). Then, means of the NDCGs across 552 patients with pathogenic mutations was computed for each tool and compared (Fig. 3):

The distributions of NDCGs were compared pairwise across tools by the Kolmogorov Smirnov two samples test. Although the NDCG for the LR-A and LR-B has higher mean value compared to the other tools, according to the Kolmorogov Smirnov results, there is no statistically significant difference between LR-A or LR-B with BS or the PS.

## Discussion

A variety of methodologies have been developed in the last years to support genomic variant interpretation in an NGS analysis. These approaches widely vary in scope and implementation. From one hand, there are tools that aim at predicting the damaging effect of a variant on the gene product, for instance a protein. On the other hand, more methods aiming at detecting disease-causing, (i.e., pathogenic) variants in a patient are being developed. Caution should be taken when using the first type of methodology to infer pathogenicity, since it has been shown that they may not be accurate^1,8,9^. While the first type of tools is mainly data-driven, meaning that they learn or infer knowledge from the huge amount of genomic data that have been published over the years, pathogenicity assessment has been standardized in 2015 in a set of rules made from ACMG/AMP experts^1^. Such guidelines can rely on previous knowledge that can be derived from data, such as past interpretation of the variant from reliable sources, and they also incorporate the results of in silico prediction tools mentioned above. Automatic tools for pathogenicity assessment can be guidelines-based, i.e. they implement the ACMG/AMP guidelines in a software/web tool^14–17,19,20^ or convert the ACMG/AMP guidelines in a probabilistic framework^21^, or they could be data-driven, such as Machine Learning approaches trained to distinguish pathogenic from benign variations^26–28,30^. Like in silico prediction tools, ML approaches provide a score, which in their case represents the probability of pathogenic classification, which can be useful to prioritize variants in a patient when no disease-causing variants (i.e., VUS) have been found according to the ACMG/AMP guidelines. The application of guidelines-based approaches should be preferred, since it would eventually lead to a standardization of genomic variant interpretation worldwide, which will decrease the number of conflicting interpretations. However, the rules provided by the ACMG/AMP experts may result in uncertain classification when the information about the variant is poor or contradictory. To depict the role of these uncertain variants (VUS) in disease development, ML and computational tools could exploit the information hidden in sequencing data previously interpreted, make inference and prioritize VUS variants. VUS prioritization would support clinicians by suggesting which uncertain variants may have a higher probability of actually being pathogenic.

This paper presents a methodology that apply both ML and the ACMG/AMP guidelines to infer variant pathogenicity and provides a comparison of different tools on public datasets. Our method has been trained on a dataset containing both confirmed pathogenic and benign variants. It is suitable for the prediction of any type of variant datasets, as well as of single variants provided by the user. Additionally, it can be used to prioritize all variants identified in patient samples as standard practice in clinical settings. The performance both in binary classification (benign vs pathogenic) and prioritization (i.e., the ability to rank the more likely pathogenic variants at the top of the list of variants for each patient) were investigated on different dataset. Tools selected for comparison with the here proposed method are: (1) the Pathogenicity Score (PS)^19^ which is a number from −1.5 to + 18 and is proportional to the number of ACMG/AMP criteria in different levels of evidence verified by the variant; (2) the Bayes Score (BS)^21^ that converts ACMG/AMP criteria in a probability of pathogenicity between 0 and 1; (3) CADD and (4) VVP, two widely-used in silico prediction tools.

The LR has been chosen as ML method to model the probability of pathogenicity of a variant given its features. Two different approaches to characterize each variant were considered. The first one, the “A” approach uses only ACMG/AMP-based features, while with the second one, the “B” approach adds additional features, such as functional effect and population allele frequency. The comparison of the two approaches provides indications about the utility of adding annotation information to increase performance, or if the ACMG/AMP features alone are sufficient.

More than 8000 variants reported to be pathogenic or benign for a broad set of disease in the Clinvitae dataset up to 2017 were used as reference data set to train the two LR models (LR-A and LR-B). In this dataset, the proportion of pathogenic variants is about 40%. However, if the scenario where the trained model is applied to classify variants in a real patient is considered, few pathogenic variants compared with (potentially thousands) benign variants are expected. Ideally, ML algorithms should be trained on a dataset that is as much as possible close to the real population. Unfortunately, few studies report a large cohort of patients with the complete genomic profile and validated variant interpretation, and they are focused on selected gene panels for specific disorders^2,46^. To cope with this disproportion in the training data and also to increase pathogenicity precision, the best threshold for classification based on the Precision-Recall Curve was selected, balancing precision and recall through the ${F}_{\beta }$ metric. Two different datasets (Clivitae Validation and the ICR639 hereditary cancer dataset^2^) were extracted to evaluate and compare different tools. The first one was filtered for variants that were included in model development, to avoid type I circularity ^47^. The proportion of benign and pathogenic variants is the same in all Clinvitae datasets (Fig. 1B,C). As shown in Fig. 1, the proportion of pathogenic/benign variants is much different between Clinvitae datasets and ICR639 dataset, representative of a real cohort of patients. Moreover, only two genes in the ICR639 panel (BRCA1 and BRCA2) are among the most represented variants in the training set. These differences can be an explanation for the lower performances achieved by the trained LR-A and LR-B on ICR639 dataset. In fact, when the distribution of variants is similar to those reported in the training set (as in Clinvitae validation dataset), LR methods outperform the guidelines-based scoring methods (PS and BS) as shown in Tables 2 and 3. No statistically significant difference is found between LR-A and LR-B in this dataset; therefore adding annotation information does not improve classification, and ACMG/AMP-based features alone are sufficiently informative. This is also confirmed by LR coefficient estimates (Table S3): ACMG/AMP features are retained, while almost half of the annotation features are actually discarded from the model.

On Clinvitae variants interpreted as VUS by the ACMG/AMP guidelines, each tool shows decreasing performance for all the metrics (Table 3). This result may confirm that VUS variants are not characterized well by the current knowledge base underlining the ACMG/AMP guidelines. However, the ML approaches show a less significant decrease in performance, maintaining an acceptable balance between precision and recall, while the Bayes Score and the Pathogenicity Score have very low recall (10% and 51%, respectively). This decrease can be explained by the nature of the BS and PS scores: since they can be seen as the conversion of ACMG/AMP levels of evidence into a numerical space, they are less suited for VUS classification. The ML approaches, on the other hand, may have learned hidden patterns from the training data that helped them in VUS classification based on the ACMG/AMP criteria profile. The LR-B (that includes the annotation features) in particular has slightly higher performances compared with LR-A (with only ACMG/AMP-based criteria).

The performance of the data-driven approaches (LR-A and LR-B) in classification significantly decreased on the ICR639 dataset, which is different in the proportion of benign and pathogenic variants (Fig. 1C) and also in the gene composition. It is worth to know that all variants reported in the ICR639 VCF files that were not interpreted as *pathogenic* were assumed to be *benign*. This assumption is necessary to apply our binary classification approaches. Additionally, we have to consider that the ICR639 dataset was collected from real cases under the assumption of monogenic inheritance hypothesis. Therefore, only one causative variant per sample is expected, assumed to be the confirmed pathogenic variant, while the remaining are likely to be benign. Yet, a proportion of the variants labeled as “benign” may be confirmed as pathogenic in the future. For this reason, it is interesting to focus the evaluation of the performance on the identification of the confirmed pathogenic variants. Moreover. it has been evaluated if variants wrongly classified belongs to a gene not properly represented in the training set. For LR-A, among the 118 genes with false positive variants (benign variants that are classified as pathogenic), 63 were not reported in the training set. For the 55 remaining genes, 22 of them have less than five variants in the training set, and the majority of the genes with more than 15 variants have a much higher proportion of pathogenic variants in the training set. Among the three genes with false negative variants (pathogenic variants reported as benign), only one gene is not reported in the training set. The other two genes are PALB2, which is represented in the training set with only two benign and one pathogenic variant, and BRIP1, which has only one benign variant reported in the training set. Despite the classification threshold has been set to minimize the number of false positive based on the Clinvitae PTT Set, on the ICR639 dataset LR-A and LR-B showed a higher number of false positive compared to PS and BS (Table 4S–7S). This evidence reinforces the need to draw on training data that are more representative of the true distributions of genomic variants in patients. However, BS predicted as benign 25 pathogenic variants, while LR-A only has one false negative (i.e., a pathogenic variant predicted as benign). CADD and VVP have very low classification performances. Moreover, it was evaluated on the ICR639 hereditary cancer dataset whether these approaches could solve variants interpreted as VUS through the ACMG/AMP guidelines. Among the guidelines-based tools, the PS score have a good balance both in pathogenic and benign detection ability, while the BS score, that showed good performances on the entire dataset, has lower ability to interpret VUS variants. This result can be explained by the different thresholding strategies that were applied to PS and BS: while in the first case the best threshold was selected with a data-driven approach, for the BS the threshold for pathogenicity was kept to 0.9, as stated by the ACMG/AMP guidelines. LR-A and LR-B have a really good ability to detect pathogenic variants that were reported as VUS, especially LR-B, which failed to interpret only two pathogenic variant and has a lower number of false positive compared to LR-A. Even if the difference in performance between LR-A and LR-B are small, it could be hypothized that adding annotation features to ACMG/AMP criteria may be able to improve classification when ACMG/AMP features alone are not informative, which is the case of VUS variants, given that the LR-A features based on ACMG/AMP criteria do not explicit variant annotation information. Within this context, our approach assigned to each variant, included VUS, a probabilistic score, providing consistent pathogenicity measurement that may support clinicians in the interpretation step. Yet, the LR-A model performs slightly better than the LR-B on Clinvitae VUS variants, proving that additional experiments with novel datasets should be performed. Moreover, it is worth to note that some of the annotation features within the B approach are implicitly used to trigger the ACMG/AMP levels of evidence withing the LR-A approach. On one hand, this may imply that the ACMG/AMP features alone are sufficient to interpret genomics variants; on the other hand, additional annotation features that are not exploited to trigger the ACMG/AMP levels of evidence may add information to depict VUS variants.

In terms of prioritization, different tools have been evaluated checking if they are able to rank pathogenic variants in the very first positions. If thousands of variants are interpreted, a good prioritization tool could be essential to ease and support variant interpretation in a clinical setting. Prioritization was tested on each ICR639 patient, and as reported in Fig. 3, all tools except for CADD and VVP have good mean performances, with LR-B and LR-A showing the highest. However, no statistically significant difference was found between LR-A, LR-B, PS and BS in prioritization.

To conclude, our model was trained on known benign and pathogenic variants associated with a broad set of diseases and provided a probabilistic pathogenicity score for each of them. This general model, as shown in the paper, can be applied also to gene panel associated with hereditary cancer with good prioritization performance and good ability to solve uncertain cases. However, the classification performance can vary if the test dataset significantly differs from the training set. Caution must be taken when interpreting prediction of a ML model that has been trained on a different population. Notably, when tested on similar data, ML incorporating ACMG/AMP-based features shows to improve pathogenicity detection compared to simply scoring methods derived from ACMG/AMP guidelines, and to solve a higher number of VUS cases. Therefore, even though in the very last years the number of interpreted variants that are available and that can be used to build data-driven approaches is increasing, it is desirable to have access to more interpreted data from single patients’ genomes. Moreover, it would be necessary to test the add value of our approach in clinical practice by evaluating whether it can support clinicians to solve specific uncertain cases.

## Supplementary Information

Supplementary Information.

## Data Availability

Source code is provided in a Jupyter Notebook available at https://github.com/GiovannaNicora/MLVar. Data are available upon request on figshare.

## Abbreviations

ACMG: American College of Medical Genetics and Genomics
AMP: American Association of Molecular Pathology
ML: Machine learning
LR: Logistic regression
PS: Pathogenicity score
BS: Bayesian score
