# Supplementary material for: A machine learning approach based on ACMG/AMP guidelines for genomic variant classification and prioritization
Source: Sci Rep. 2022 Feb 15;12:2517. doi: 10.1038/s41598-022-06547-3 (PMC8847497; doi:10.1038/s41598-022-06547-3)
Supplement: Supplementary file 1 — Supplementary Information. [file 41598_2022_6547_MOESM1_ESM.pdf]

Supplemental Material

Feature Name	Description	Source	Type	Approac h
nPVS	Number of criteria triggered by the variant which fall in ACMG/AMP “Very Strong” pathogenic level of evidence	eVai	Integer	A-B
nPS	Number of criteria triggered by the variant which fall in ACMG/AMP “Strong” pathogenic level of evidence	eVai	Integer	A-B
nPM	Number of criteria triggered by the variant which fall in ACMG/AMP “Moderate” pathogenic level of evidence	eVai	Integer	A-B
nPP	Number of criteria triggered by the variant which fall in ACMG/AMP “Supporting” pathogenic level of evidence	eVai	Integer	A-B
nBA	Number of criteria triggered by the variant which fall in ACMG/AMP “Stand-Alone” benign level of evidence	eVai	Integer	A-B
nBS	Number of criteria triggered by the variant which fall in ACMG/AMP “Very Strong” benign level of evidence	eVai	Integer	A-B
nBP	Number of criteria triggered by the variant which fall in ACMG/AMP “Supporting”	eVai	Integer	A-B

	benign level of evidence			
RepeatMasker	variant occurs in a region where DNA short sequences are repeated	http://www.repeatmasker.org	Boolean	B
Exac_AF	ExAC frequency of the ALT allele	ExAC version r0.3	Float 0-1	B
Exac_isTarget	The genomic locus is covered by ExAC according to the WES design file (.bed)	ExAC version r0.3	Boolean	B
gnomAD_WGS_ gnomAD_WES_AF_ALL		gnomAD		B
gnomAD_WGS_gnomAD_ WES_Hom_ALL		gnomAD		B
dbSNP_1TGP_ALT_freq	1000 Genomes Project ALT allele frequency [0-1] as reported in dbSNP	dbSNP version 147	Float 0-1	B
ESP_All_Freq	ALT allele frequency in ESP general population	ESP 6500SIv2	Float 0-1	B
DANN_score	Probability for this variant (SNV only) to be deleterious according to DANN score. Both for coding and non-coding genomic variants.	DANN[1]	Float 0-1	B
dbscSNV_AB_score	Probability for this variant (SNV only) to be deleterious for the nearby splicing site. Score computed by AdaBoost machine learning classifier. Valid for variants at −3 to+8at the 5’splice site and −12 to+2at the 3’ splice site	dbscSNV	Float 0-1	B
dbscSNV_RF_score	Probability for this variant (SNV only) to be deleterious for	dbscSNV	Float 0-1	B

	the nearby splicing site. Score computed by Random Forest machine learning classifier. Valid for variants at −3 to +8 at the 5’ splice site and −12 to +2 at the 3’ splice site			
PaPI_score	PaPI (http://papi.unipv.it) score for this variant to be damaging/tolerated for the protein structure/function. It is the combined score given by PolyPhen-2, SIFT and PseeAC-RF classifiers	PaPI	Float 0-1	B
PolyPhen-2 score	PolyPhen-2 (HumVar) score for this variant to be damaging/tolerated for the protein structure/function	PolyPheno-2	Float 0-1	B
SIFT_score	SIFT score for this variant to be damaging/tolerated for the protein structure/function	SIFT	Float 0-1	B
PseeAC-RF score	Random Forest Pseudo-Amino acidic classifier score for this variant to be damaging/tolerated for the protein structure/function	PseeAC	Float 0-1	B
Hotspot	Whether the variant occurs in a ClinVar hotspot region	eVai	Boolea n	B
Effect_columns	Percentage of transcripts in which the variant has a particular effect. For instance, frameshift_variant= 0.5 means that the	Transcript-variant effect according to the MISO[2] sequence ontology terms	Float 0-1	B

	variant is frameshift in half of the transcript in which it occurs			

Table S1: List of features for each variant, along with description, type and whether the feature is exploited in the A or A+B approach.

Feature Type	Feature	Beta
**ACMG/AMP-based**	nPVS	3.24
	nPS	8.41
	nPM	9.41
	nPP	5.22
	nBA	-2.28
	nBS	-1.25
	nBP	-6.1

Table S2: Logistic Regression A (LR-A) approach: coefficients estimated

Feature Type		Feature	Beta
**ACMG/AMP-based**	1	nPVS	2.46
	2	nPS	8.63
	3	nPM	9.58
	4	nPP	3.43
	5	nBA	-0.22
	6	nBS	-1.16
	7	nBP	-4.83
**Annotation (Repeated region)**	8	RepeatMasker	-0.46
**Annotation (Population Frequency)**	**9**	**Exac_AF**	**0**
	10	Exac_isTarget	-1.91
	**11**	**gnomAD_WGS_ gnomAD_WES_AF_ALL**	**0**
	12	gnomAD_WGS_gnomAD_ WES_Hom_ALL	- 0.0015
	13	dbSNP_1TGP_ALT_freq	-0.47
	**14**	**ESP_All_Freq**	**0**
**Annotation (*in-silico* prediction)**	15	DANN_score	-0.59
	**16**	**dbscSNV_AB_score**	**0**
	17	dbscSNV_RF_score	2.56

	18	PaPI_score	0.47
	19	PolyPhen-2 score	0.53
	20	SIFT_score	-0.02
	21	PseeAC-RF score	0.66
	22	Hotspot	-1.47
**Annotation (effect type)**	23	stop_gained	1.8
	24	stop_lost	-0.72
	25	frameshift_variant	0.48
	**26**	**Start_loss**	**0**
	**27**	**Exon_loss**	**0**
	**28**	**Exon_loss_variant**	**0**
	29	Splice_acceptor_variant	1.65
	30	Splice_donor_variant	0.72
	31	disruptive_inframe_insertion	-1.37
	32	disruptive_inframe_deletion	-2.02
	**33**	**Inframe_insertion**	**0**
	34	Inframe_deletion	-2.38
	35	Missense_variant	-0.68
	**36**	**Initiator_codon_variant**	**0**
	37	Splice_region_variant	0.411
	**38**	**Start_retained**	**0**
	**39**	**Non_canonical_start_codon**	**0**
	**40**	**Stop_retained_variant**	**0**
	41	Synonymous_variant	-1.86
	**42**	**Exon_variant**	**0**
	**43**	**transcript**	**0**
	44	Intron_variant	-0.81
	**45**	**5_prime_UTR_premature_start_codon_gain_variant**	**0**
	**46**	**3_prime_UTR_truncation**	**0**
	**47**	**5_prime_UTR_truncation**	**0**
	48	5_prime_UTR_variant	-0.904
	**49**	**3_prime_UTR_variant**	**0**
	50	Intragenic_variant	-1.78
	**51**	**Intergenic_region**	**0**
	**52**	**Upstream_gene_variant**	**0**
	**53**	**Downstream_gene_variant**	**0**

Table S3: Logistic regression B (LR-B) approach: coefficients estimates. Features written in red have estimated betas equal to zero.

Metrics for Classification Performance

Several metrics are reported for comparing tool performances. These metrics are computed based on the confusion matrix, that collects raw counts of correctly and incorrectly classified variants known to be pathogenic or benign. We indicate as Positive those variants known to be Pathogenic, while the Negative class is composed of benign variants. Therefore, the confusion matrix for a tool on a given dataset is the following:

	**Benign (Negative)**	**Pathogenic (Positive)**
**Predicted Benign**	TN	FN
**Predicted Pathogenic**	FP	TP

where:

- TN (True Negative) is the number of benign variants correctly classified as benign from the tool.
- FP (False Positive) is the number of benign variants incorrectly classified as pathogenic from the tool.
- TP (True Positive) is the number of pathogenic variants correctly classified as pathogenic from the tool.
- FN (False Negative) is the number of pathogenic variants incorrectly classified as benign from the tool.

From the confusion matrix, it is possible to compute several metrics such as:

- $Accuracy = \frac{TP+TN}{TP+TN+FN+FP}$, that is the proportion of correctly classified examples in a test set. Accuracy is a widely used metrics, but it can lead to misinterpreted results when classes are imbalanced, i.e. when the number of TN is much greater than the number of TP, or vice-versa.
- $Recall = \frac{TP}{TP+FN}$. The recall, or sensitivity, is the proportion of positive instances correctly classified (in our case, it represents the ability to correctly identify pathogenic variants).
- $Specificity = \frac{TN}{TN+FP}$ which is the proportion of benign variants correctly identified
- $Precision = \frac{TP}{TP+FP}$ measures the fraction of variants that are actually pathogenic among all the predicted pathogenic variants

- $F1 = \frac{2TP}{2TP+FP+FN}$ which represents the harmonic mean between precision and recall
- $Balanced\ Accuracy = \frac{Recall+Specificity}{2}$
- $Matthews\ Correlation\ Coefficient\ (MCC) = \frac{TP \times TN - FP \times FN}{\sqrt{(TP+FP)(TP+FN)(TN+FP)(TN+FN)}}$: this metric is more reliable when dealing with unbalanced dataset compared to the F1 score or accuracy [3].
- ROC AUC (Receiver Operating Curve Area Under the Curve) is the area under the curve that illustrates different values of true positive rate against the false negative rate when different thresholds for classification are used. A perfect ROC has AUC close to 1.
- PRC AUC (Precision-Recall Area Under the Curve): area under the curve computed for different values of precision and recall when classification threshold varies. The PRC is more informative when the dataset in imbalanced [4].

F_β measure approach

Machine learning classifiers, such as the Logistic Regression, compute the probability that an instance belongs to a class given its attributes' profile. In our case, the Logistic Regression gives us the probability that a variant is pathogenic given the variant's features (A or B approach). Since the classification problem is binary, the probability that the variant is benign will be 1 minus the pathogenic probability. Probabilities are translated into the binary classification by simply putting a threshold: usually, if a variant has pathogenic probability equal or greater than the benign probability then the predicted class is "Pathogenic". This means that the threshold for classification is 0.5, and the two classes have the same weight. However, in some cases we may want to be more precise in detecting one of the two classes. For instance, in a population screening for a severe pathology that can be easily treated at the initial stage, we want to detect the higher number of positives as possible, even if this would lead to increase the number of False Positive. Classification threshold can be adjusted also to deal with imbalanced dataset, where the number of instances in one class is much greater than the number of instances in the other class [5].

We changed the classification thresholds based on the following considerations: in our specific case, we are training ML based on a dataset (Clinvitae Training) which is not highly imbalanced but has a much higher proportion of pathogenic variants compared to a real case scenario, as shown in Fig. 1A and Fig. 1C. In fact, since a patient usually harbors very few pathogenic variants, we want to assure that the model applied to a real case will not provide a high number of False Positive, that may slow the screening process made by the user. Therefore, we want to be *precise* in pathogenicity detection

(see the definition of precision above). The F_β combines precision and recall in a single measure that can weights the two terms through the β factor [6]:

$$F_\beta = \frac{(\beta^2 + 1) \times Precision \times \ Recall}{\beta^2 \times Precision + Recall}$$

When $\beta = 1$ then recall and precision are equally important. For $\beta > 1$, recall weight more than precision, while for $\beta < 1$ precision weights more. For instance, if $\beta = 0.5$ precision weights twice the recall. We chose $\beta = 0.35$, and we calculate the corresponding $F_{0.5}$ for different thresholds value. Then we chose the threshold corresponding to the highest value of $F_{0.5}$, which for LR-A corresponds to 0.865 and for LR-B to 0.79. With these thresholds, the values of precision and recall for LR-A and LR-B on Clinvitae Probability Tuning Set are shown on the Precision-Recall Curves (PRC) reported in Figure S1.

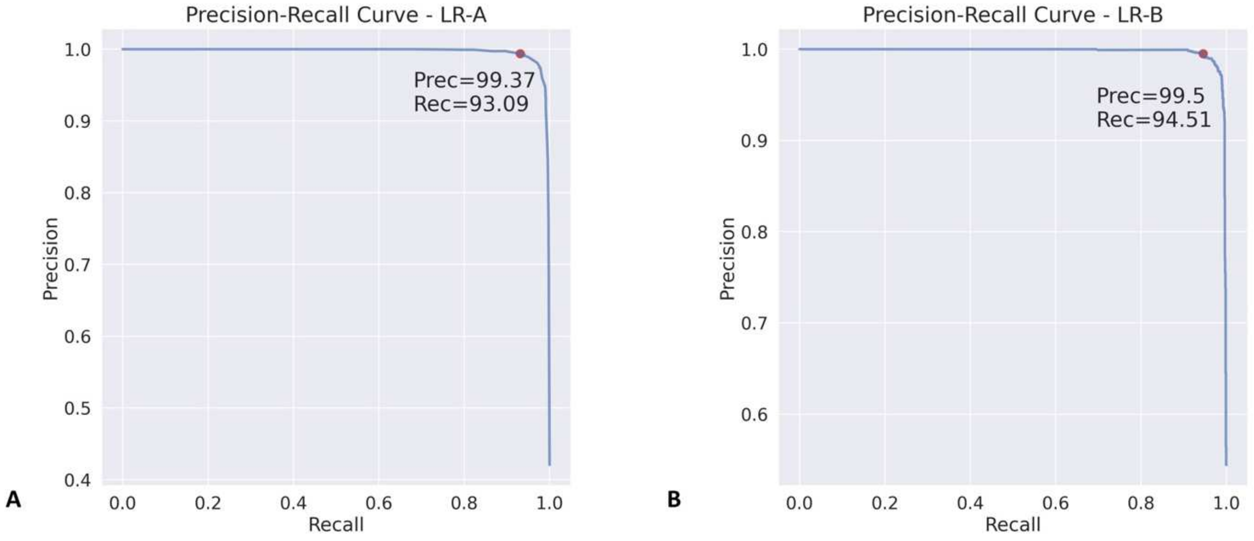

Figure S1: A) PRC of LR-A on Clinvitae Probability Set. B) PRC of LR-B on Clinvitae Probability Test Set. Red circles represent the values of precision and recall for the selected thresholds.

	LR-A	LR-B	PS	BS	CADD	VVP
**Accuracy**	0.9738	0.9814	0.9827	0.9906	0.9338	0.6027
**Precision**	0.5382	0.6227	0.6407	0.7859	0.3082	0.071
**AUC**	0.9856	0.9886	0.9858	0.9731	0.9368	0.7933
**F1**	0.6993	0.7664	0.7776	0.8620	0.4643	0.1326
**Recall**	0.9981	0.9963	0.9890	0.9545	0.94	0.9963
**Balanced Accuracy**	0.9856	0.9886	0.9858	0.9731	0.9368	0.7933
**MCC**	0.7229	0.7801	0.7888	0.8616	0.5172	0.2038
**PRC**	0.5373	0.6205	0.6340	0.7515	0.2916	0.070

Table S4 Results of Logistic Regression A approach (LR-A), Logistic Regression B approach (LR-B), Pathogenicity score (PS), the Bayesian approach (BS), CADD and VVP on the ICR639 validation set

	Benign	Pathogenic
**Predicted Benign**	170425	1
**Predicted Pathogenic**	471	549

Table S5: Confusion Matrix of LR-A on ICR639 variants

	Benign	Pathogenic
**Predicted Benign**	17164	2
**Predicted Pathogenic**	332	548

Table S6: Confusion Matrix of LR-B on ICR639 variants

	Benign	Pathogenic
**Predicted Benign**	17191	6
**Predicted Pathogenic**	305	544

Table S7: Confusion Matrix of PS on ICR639 variants

	**Benign**	**Pathogenic**
**Predicted Benign**	17353	25
**Predicted Pathogenic**	143	525

Table S8: Confusion Matrix of BS on ICR639 variants

	**Benign**	**Pathogenic**
**Predicted Benign**	11521	1
**Predicted Pathogenic**	315	25

Table S9: Confusion Matrix of LR-A on ICR639 variants predicted as VUS according to the ACMG/AMP guidelines

	**Benign**	**Pathogenic**
**Predicted Benign**	11684	2
**Predicted Pathogenic**	188	24

Table S10: Confusion Matrix of LR-B on ICR639 variants predicted as VUS according to the ACMG/AMP guidelines

	**Benign**	**Pathogenic**
**Predicted Benign**	11689	6
**Predicted Pathogenic**	147	20

Table S11: Confusion Matrix of PS on ICR639 variants predicted as VUS according to the ACMG/AMP guidelines

	**Benign**	**Pathogenic**
**Predicted Benign**	11816	19
**Predicted Pathogenic**	20	7

Table S12: Confusion Matrix of BS on ICR639 variants predicted as VUS according to the ACMG/AMP guidelines

	**Benign**	**Pathogenic**
**Predicted Benign**	11088	7
**Predicted Pathogenic**	748	19

Table S13: Confusion Matrix of CADD on ICR639 variants predicted as VUS according to the ACMG/AMP guidelines

	**Benign**	**Pathogenic**
**Predicted Benign**	5494	0
**Predicted Pathogenic**	6342	26

Table S14: Confusion Matrix of VVP on ICR639 variants predicted as VUS according to the ACMG/AMP guidelines

References

1. Quang, D., Chen, Y. & Xie, X. DANN: a deep learning approach for annotating the pathogenicity of genetic variants. *Bioinforma. Oxf. Engl.* **31**, 761–763 (2015).
2. Eilbeck, K. *et al.* The Sequence Ontology: a tool for the unification of genome annotations. *Genome Biol.* **6**, R44 (2005).
3. Chicco, D. & Jurman, G. The advantages of the Matthews correlation coefficient (MCC) over F1 score and accuracy in binary classification evaluation. *BMC Genomics* **21**, (2020).
4. Saito, T. & Rehmsmeier, M. The Precision-Recall Plot Is More Informative than the ROC Plot When Evaluating Binary Classifiers on Imbalanced Datasets. *PLoS ONE* **10**, (2015).
5. Zou, Q., Xie, S., Lin, Z., Wu, M. & Ju, Y. Finding the Best Classification Threshold in Imbalanced Classification. *Big Data Res.* **5**, 2–8 (2016).
6. Chinchor, N. MUC-4 Evaluation Metrics. in *Proceedings of the 4th Conference on Message Understanding* 22–29 (Association for Computational Linguistics, 1992). doi:10.3115/1072064.1072067.
